# Analysis of Viral Diversity in Relation to the Recency of HIV-1C Infection in Botswana

**DOI:** 10.1371/journal.pone.0160649

**Published:** 2016-08-23

**Authors:** Sikhulile Moyo, Alain Vandormael, Eduan Wilkinson, Susan Engelbrecht, Simani Gaseitsiwe, Kenanao P. Kotokwe, Rosemary Musonda, Frank Tanser, Max Essex, Vladimir Novitsky, Tulio de Oliveira

**Affiliations:** 1 Division of Medical Virology, Stellenbosch University, Tygerberg, South Africa; 2 Botswana-Harvard AIDS Institute Partnership, Gaborone, Botswana; 3 Wellcome Trust Africa Centre for Health and Population Studies, Dorris Duke Medical Research Centre, Nelson R Mandela School of Medicine, University of KwaZulu-Natal, Durban, South Africa; 4 National Health Laboratory Services (NHLS), Tygerberg Coastal, South Africa; 5 Department of Immunology and Infectious Diseases, Harvard T.H. Chan School of Public Health, Boston, Massachusetts, United States of America; 6 Research Department of Infection, University College London, London, United Kingdom; 7 College of Health Sciences, University of KwaZulu-Natal, Durban, South Africa; "INSERM", FRANCE

## Abstract

**Background:**

Cross-sectional, biomarker methods to determine HIV infection recency present a promising and cost-effective alternative to the repeated testing of uninfected individuals. We evaluate a viral-based assay that uses a measure of pairwise distances (PwD) to identify HIV infection recency, and compare its performance with two serologic incidence assays, BED and LAg. In addition, we assess whether combination BED plus PwD or LAg plus PwD screening can improve predictive accuracy by reducing the likelihood of a false-recent result.

**Methods:**

The data comes from 854 time-points and 42 participants enrolled in a primary HIV-1C infection study in Botswana. Time points after treatment initiation or with evidence of multiplicity of infection were excluded from the final analysis. PwD was calculated from quasispecies generated using single genome amplification and sequencing. We evaluated the ability of PwD to correctly classify HIV infection recency within <130, <180 and <360 days post-seroconversion using Receiver Operator Characteristics (ROC) methods. Following a secondary PwD screening, we quantified the reduction in the relative false-recency rate (rFRR) of the BED and LAg assays while maintaining a sensitivity of either 75, 80, 85 or 90%.

**Results:**

The final analytic sample consisted of 758 time-points from 40 participants. The PwD assay was more accurate in classifying infection recency for the 130 and 180-day cut-offs when compared with the recommended LAg and BED thresholds. A higher AUC statistic confirmed the superior predictive performance of the PwD assay for the three cut-offs. When used for combination screening, the PwD assay reduced the rFRR of the LAg assay by 52% and the BED assay by 57.8% while maintaining a 90% sensitivity for the 130 and 180-day cut-offs respectively.

**Conclusion:**

PwD can accurately determine HIV infection recency. A secondary PwD screening reduces misclassification and increases the accuracy of serologic-based assays.

## 1.0 Background

Identification of HIV infection recency is crucial for the accurate estimation of HIV incidence, the evaluation of the effectiveness of antiretroviral treatment (ART) programs, and the timely linking of HIV-infected individuals (and their partners) to treatment and care services [1–9]. The timing of infection can also be used to identify the immunological and virological characteristics of individuals who have recently acquired HIV and to characterize individuals who are putative transmitters in linked infections [10–14].

The longitudinal cohort design is currently recognized as the standard approach to identify new HIV infections [15–17]. However, frequent HIV testing at the population level is a logistically challenging, time-consuming, and expensive enterprise. For these reasons, large-scale surveillance programs are typically undertaken on a periodic basis of 12 or more months, making it difficult to ascertain the precise date of an HIV infection. Factors associated with illness, work commitments, temporary or cyclical migration, assumed knowledge of current HIV status, and the stigma associated with a positive status, among others, may decrease the frequency at which an eligible individual is captured for HIV testing [18–20]. On the other hand, the identification of new HIV infections is possible for experimental trials where relatively small cohorts (typically <500 individuals) are routinely tested on a weekly or monthly basis [21–23].

There is growing scientific interest in the use of cross-sectional sampling methods to identify individuals recently infected with HIV. Cross-sectional methods can mitigate the impact of infrequent testing and the high lost-to-follow-up rates that are associated with the longitudinal approach [24–28]. Biomarker data collected from cross-sectional sampling has also shown great promise in the ability to differentiate between recent and established HIV infections. Serological assays, for example, the Calypte Incidence Assay (BED) and Limiting Antigen assay (LAg), depend on the markers of evolution of the host immune response to HIV, such as antibody levels, avidity, isotype and proportion [29–35]. Attention is now turning to the improvement of assay-based methods and the use of multi-assay algorithms (MAA) to better predict HIV infection recency [36].

One area that is receiving increasing attention is the use of a viral diversity measure [11, 13, 37–41]. The majority of HIV infections are caused by the transmission of a single founder virus, resulting in a relatively homogeneous population of viral quasispecies during the early stage of HIV infection [42–45]. Due to the error prone nature of the Reverse Transcriptase (RT) enzyme and the host immune response to pressure, the virus is able to diversify rapidly over time. The approximately linear diversification of HIV in early infection [46] provides a rationale for using viral diversity as a marker for HIV infection recency [11, 39, 47, 48]. One example of a time-dependent, viral-based diversity measure is the pairwise nucleotide diversity (PwD). PwD measures the average number of pairwise nucleotide differences per site in DNA sequences [11, 37, 38, 43, 49, 50]. Assays based on a measure of PwD should be less sensitive to the variability in immune responses modulated by HIV clade, host genetics and routes of transmission. However, viral-based assays are more challenging and costly to implement.

About 20–25% of HIV infections are caused by the transmission of multiple viral variants [43, 51–53]. The rate of HIV-1 super-infection could be comparable with the rate of primary HIV-1 infection [54], although super-infection is less frequent in the HIV-1C epidemic in South Africa [55]. Ignoring multiplicity of HIV infection could mislead analysis and lead to erroneous conclusions due to increased intra-host diversity in cases with multiple transmitted HIV variants, or in super-infection. Using intra-host viral sequences that represent HIV quasispecies provides an opportunity to identify phylogenetically distinct viral lineages and take into account multiplicity of HIV infection.

In this paper, we use data from a frequently tested longitudinal HIV-1C infection cohort (the “*Tshedimoso*” study from Botswana) for which the exact date of infection is known. We assess the accuracy of the PwD assay to correctly classify HIV infection recency, and compare its performance with the BED and LAg assays. Because of the high cost currently associated with single genome sequencing, we further investigate the use of a MAA (BED plus PwD or LAg plus PwD) to increase accuracy and maintain affordability. We also evaluate the addition of viral load (VL) as a covariate to the MAA algorithm. We discuss the potential of cross-sectional, biomarker information and the use of MAAs as an affordable and accurate alternative to the longitudinal cohort approach.

## 2.0 Methods

### 2.1 Participants and specimens

The data comes from 854 time points and 42 study participants enrolled into a primary HIV-1 subtype C infection longitudinal cohort in Botswana (the “Tshedimoso” study) from April 2004 to April 2008 [56, 57]. Recent HIV-1 infections were identified by a positive HIV-1 RNA test combined with a negative HIV-1 serology in double enzyme immunoassay [58] or by applying a 2-step testing algorithm using the Vironostika HIV-1 Plus O Microelisa System (bioMérieux, Durham, NC) [59]. Acutely infected participants had weekly visits for the first 2 months, biweekly visits for the next 2 months and monthly visits for the first year following the date of seroconversion. Participants were then followed-up on a quarterly basis after the first post-seroconversion year. The study design and participant characteristics are described in greater detail elsewhere [56, 59, 60]. This study was conducted according to the principles expressed in the Declaration of Helsinki. The study was approved by the Institutional Review Boards of Botswana and the Harvard School of Public Health. All patients provided written informed consent for the collection of samples and subsequent analysis.

### 2.2 Serological assays and HIV pairwise diversity for recency determination

Blood specimens from 42 participants were used to generate 594 BED (Calypte Aware BED HIV-1 Incidence Test, Calypte Biomedical Corporation; Portland, USA) and 597 LAg (Limiting Antigen Assay, Sedia BioSciences; Portland, USA) test results according to manufacturers’ instructions [34, 35]. All available specimens were included for testing with both serological assays. UNAIDS/WHO guidelines for determining infection recency recommend the removal of specimens with evidence of ART use [33, 61, 62], resulting in the exclusion of 49 time points and one participant from our analysis (see S1 Fig of the Supplement).

The intra-host viral sequences representing HIV-1C quasispecies were generated by single genome amplification and sequencing, as described elsewhere [47, 63]. The primary goal of sequencing was analysis of viral diversity and evolution during primary HIV-1C infection [47, 63]. The quarterly time points spanning the period from the earliest sample at enrollment to about 500 days post seroconversion were selected from the available sampling points (S4 Fig). Individuals with acute HIV-1C infection were sampled more frequently than individuals enrolled during Fiebig stages IV-V [64].

The targeted region spanned HIV-1C *env* gp120 V1C5 corresponding to nucleotide positions 6,615 to 7,757 of HXB2. A total of 2,540 single genome amplification sequences were generated from an average of 6 time points per patient and an average of 10 multiple-sequences (quasispecies) per time point. Both viral RNA and proviral DNA were used as templates for amplification and sequencing. Viral sequences were codon-aligned by muscle [65] in MEGA 6.06 [66]. Mean pairwise distances (PwD) were estimated per participant per time point using the Maximum Composite Likelihood model and pairwise deletion of gaps in MEGA 6.06 [66]. The accession numbers of the viral sequences used in this study are KC628761—KC630726.

### 2.3 Multiplicity of HIV infection

Previous research has shown that multiplicity of infection can result in highly variable PwD values [33, 61, 62]. For this reason, we undertook a phylogenetic analysis to identify and exclude time points with multiple founder variants or potential super-infection. Multiplicity was determined by the branching topology of viral quasispecies (~1,200 bp V1C5 region of HIV-1 env gp120) derived from a single time point of sampling. A total of 2,540 viral sequences from 42 subjects were analyzed with 1322 HIV-1 subtype C V1C5 sequences retrieved from the Los Alamos National Laboratory (LANL) HIV Database (S2 Table). Phylogenetic trees were inferred by the Maximum-Likelihood (ML) using Fasttree v.2.1.8 with a GTR model of nucleotide substitution [67]. Phylogenetic trees were visualized and inspected in FigTree [68]. Monophyletic clustering was interpreted as HIV transmission from a single source including transmission of multiple viral variants from the same source. We excluded 47 time points and one participant with viral quasispecies separated by reference sequence(s), as these were interpreted as HIV transmissions from multiple sources (including potential super-infection). The final sample size was 758 time-points from 40 participants (see S1 Fig of the Supplement).

### 2.4 Statistical Analysis

We used a receiver operating characteristics (ROC) analysis to compare the accuracy of the BED, LAg and PwD assays to identify HIV infection recency. Frequent and repeated testing of study participants enabled us to identify the *known* instances of a recent HIV infection. Specifically, known HIV infection recency was defined as any specimen obtained within a <130, <180 or <360-day post-seroconversion period. For each BED, LAg and PwD assay, we then classified a specimen as a “recent” infection if it was below a threshold value, or classified the specimen as an “established” infection if it was above this threshold. We refer to these as the *classified* instances of a recent HIV infection [69]. For example, we classified specimens with a BED value ≤0.8 as a recent infection or an established infection otherwise. The recommended threshold values for the BED and LAg assays are 0.8 and 1.5 respectively [34, 70].

The best performing thresholds for the PwD assay have yet to be definitively established. Previous research has suggested that the rate of increase in the pairwise sequence diversity of the HIV-1 *env* gene region is a constant rate of approximately 0.01 per year during early infection [46]. We therefore used these biological guidelines to select PwD thresholds of 0.004, 0.005 and 0.01 for the 130, 180 and 360-day cut-offs respectively. For each threshold, we obtained the sensitivity (recent infections correctly identified) and the specificity (established infections correctly identified) using maximum likelihood estimates from a logistic regression analysis. Because repeated measurements were taken for each participant over time, we calculated the standard errors and 95% confidence intervals (CI) for these estimates using the Huber-White sandwich estimator [71, 72]. Given the evaluation of multiple test thresholds, we used the highest percentage of specimens correctly classified (CC) as a guide to evaluate the performance of each PwD threshold. The CC is computed as the sum of the recent and established specimens correctly classified divided by the total number of specimens classified.

We next evaluated the predictive performance of combination BED plus PwD screening to determine infection recency, and repeated this procedure for combination LAg plus PwD screening. Specifically, our aim was to determine whether the more affordable BED or LAg assay can be combined with the more sensitive PwD assay to reduce the likelihood of a false-recent classification. We first screened for recent infections using a recommended BED threshold of 0.8 for the 180-day cut-off and a recommended LAg threshold of 1.5 for the 130-day cut-off. The threshold and cut-off combination selected for the analysis are based on the work of Kassanjee et al. and Duong et al. [33–35]. We then used the PwD assay with a threshold of 0.005 to reduce the false-recency rate associated with the primary BED and LAg screening assays. Shaw et al. [73] propose to obtain the relative true-recent rate (rTRR) and the relative false-recent rate (rFRR) of the combined BED (or LAg) and PwD assays with:

$$rTRR=\frac{P(BED=+,\;PWD=+|\;R)}{P(BED=+|R)}\;\text{and}$$

$$rFRR=\frac{P(BED=+,\;PWD=+|\;\bar{R})}{P(BED=+|\bar{R})}.$$

In the above equations, *BED*+ and *PwD*+ are the specimens classified as recent infections by the respective assay, *R* denotes the specimens known to be recent infections and $\bar{R}$ denotes the specimens known to be established infections. When considering the use of a second marker to improve predictive performance, it is expected that a high rTRR (sensitivity) is maintained while the rFRR is reduced, such that the rTRR will be close to 1.0 and the rFRR will be substantially less than 1.0 [73]. We evaluate the percentage reduction in the rFRR by the PwD assay at rTRR (sensitivity) levels of 75%, 80%, 85% and 90%. Further, we show how the addition of viral load (VL) information can improve accuracy. Research has shown that VL measurements <1,000 copies/mL are associated with false-recent infections and can identify individuals with viral suppression [74, 75]. We used the methods of Shaw et al. [73], Janes et al. [76] and Pepe et al. [77] to obtain estimates for the rFRR and its 95% confidence intervals. Statistical analyses were undertaken in Stata 13.1.

The mean duration of recent infection (MDRI), the average time being recent while infected for less than time cut-off time (*T*) was estimated using the Incidence Estimation Tools version 1.0.5.9001 (The *inctools* package in R software version 3.2.4). The *T* value of 2 years and time points with viral load above 1,000 copies/mL were used for the MDRI calculation.

## 3.0 Results

All of the 2,540 sequences from the 42 participants in the cohort were classified as subtype C. To account for multiplicity of HIV infection and avoid inflated estimate of HIV pairwise distances, time points with phylogenetically distinct viral lineages (n = 47) were excluded from analysis (see section 2.3 Multiplicity of HIV infection in Methods). The final analytic sample consisted of 758 (BED = 554, LAg = 579, and PwD = 238) time-points from 40 participants (see S1 Fig of the Supplement for the data flow diagram). Among the study participants, 28 (70%) were female. The median (IQR) age at enrollment was 27 (20–56) years. Participants were followed for a median (IQR) of 45.9 (32.4–53.9) months, with a median (IQR) of 21 (18–27) time points per participant. The mean (SD) and median (IQR) time between tests were 2.0 (±2.9) months and 1.1 (0.92–3.0) months respectively. Table 1 shows the summary statistics for the participant characteristics and covariate measures.

**Table 1:** Participant and covariate characteristics.

Participant Characteristics	n = 40	
Female, N (%)	28	(70)
Age (years), Median (IQR)	27	(20–56)
Time under observation (months), Median (IQR)	45.9	(32.4–53.9)
Difference between time points (months), Median (IQR)	1.1	(0.92–3.0)
Total time points per participant, Median (IQR)	21	(18–27)
Assay time points per participant, Median (IQR)		
BED	14	(10–19)
Lag	14.5	(7–22)
PwD	5	(4–6)
CD4 cells/μl, Median (IQR)	417	(302–569)
Viral load (log_10_) copies/mL, Median (IQR)	3.9	(2.65–4.73)

We present the maximum likelihood estimates for the PwD assay in S1 Table of the supplement. Given that there is currently no recommended PwD threshold, we show the sensitivity and specificity estimates for values ranging from 0.0005 to 0.015. For the 130-day cut-off, a PwD threshold of 0.004 gives a sensitivity of 76.2% and a specificity of 79.7%, with 77.8% of the total specimens correctly classified. For the 180-day cut-off, a PwD threshold of 0.005 gives a sensitivity of 74.5% and a specificity of 75.5%, with 74.9% of the total specimens correctly classified. We found that PwD values of 0.0055 and 0.006 performed slightly better than the 0.004 and 0.005 values for both the 130 and 180-day cut-offs, and are biologically plausible given that HIV is known to evolve at a rate of approximately 0.01 per year.

We found the PwD threshold values (reported above) to be more accurate than the recommended LAg = 1.5 and BED = 0.8 threshold values in identifying infection recency. For a 130-day cut-off and a threshold value of 1.5, the LAg assay gives a sensitivity of 71.3% and a specificity of 72.9%, with 72.4% of the total specimens correctly classified. For a 180-day cut-off and a threshold value of 0.8, the BED assay gives a sensitivity of 87.4% and a specificity of 50.2%, with 65.5% of the total specimens correctly classified. For these cut-offs and thresholds, we see that the PwD assay has a higher proportion of specimens correctly classified when compared with the LAg and BED assays.

**Fig 1:**
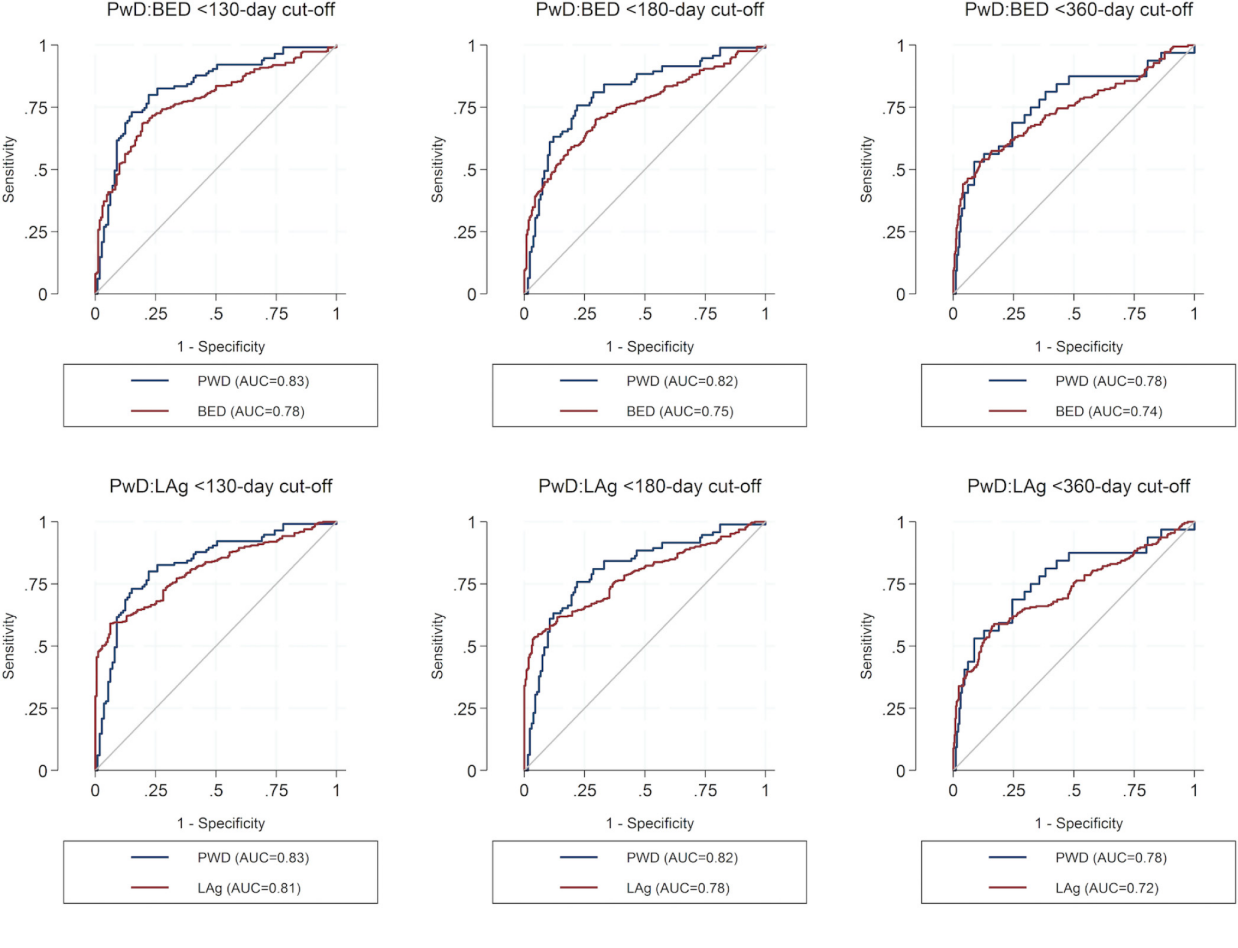
ROC graphs comparing the predictive performance of the PwD, LAg and BED assays for determing HIV infection recency for the 130, 180, and 360-day cut-offs. We used the area under the curve (AUC) of a receiver operator characteristics (ROC) graph to assess the accuracy of the PwD, BED and Lag assays to identify HIV infection recency. The best possible AUC value is 1.0. The ROC graphs are produced by calculating the sensitivity and specificity at different thresholds, which are typically incremented by a fixed value over the minimum and maximum range of the assay. The AUC results show that the PwD assay is the most accurate identifier of infection recency for the three cut-off periods.

We also compare the accuracy of the three assays to identify infection recency using the AUC estimate of a ROC graph. An AUC closer to 1.0 indicates a better accuracy, and we show these estimates along with their standard errors and 95% CIs in Table 2. The AUC value for the 130-day cut-off is 0.83 compared with 0.78 for the BED assay and 0.81 for the LAg assay. For the 180-day cut-off, these values are PwD = 0.82, BED = 0.75, and LAg = 0.79 and for the 360-day cut-off these are PwD = 0.78, BED = 0.74, and LAg = 0.72 (see also Fig 1).

**Table 2:** Area under the curve (AUC) of a receiver-operator characteristics (ROC) graph comparing the accuracy of the PwD, BED, and LAg assays in identifying HIV infection recency.

Assay	AUC	SE	95% CI
*130-day cut-off*			
PWD	0.83	0.03	0.78–0.88
BED	0.78	0.02	0.74–0.82
LAg	0.81	0.02	0.78–0.85
*180-day cut-off*			
PWD	0.82	0.03	0.76–0.88
BED	0.75	0.02	0.71–0.79
LAg	0.79	0.02	0.75–0.82
*360-day cut-off*			
PWD	0.78	0.05	0.68–0.89
BED	0.74	0.03	0.69–0.79
LAg	0.72	0.02	0.68–0.77

The table shows the results for the area under the curve (AUC) of a receiver operating characteristics (ROC) graph. The AUC is an objective measure of the accuracy of a classification schema. The best possible value is 1.0, which represents a 100% sensitivity and 100% specificity of the assay to correctly distinguish recent from established HIV infections. The results show that the PwD assay has the best predictive performance for the three window periods. CI: confidence interval.

We investigated whether MAA could further distinguish recent from established infections. Table 3 shows the ability of the PwD assay to improve predictive accuracy by reducing the relative false-recent rate (rFRR) of the LAg and BED assays. Here, we are specifically interested in the percentage reduction in the rFRR and so we subtract the rFRR estimate from 100%. As an example, we interpret the result for the LAg plus PwD combination screening for the 130-day cut-off as follows: The PwD assay reduces the rFRR by (100–48) 52% while maintaining a 90% rTRR (sensitivity) of the LAg assay. We can also interpret this result using the upper bound of the 95% CI: the PwD assay reduces the rFRR by *at least* (100–87.5) 12.5% while maintaining a LAG sensitivity of 90%.

**Table 3:** Combination assay screening to identify HIV infection recency for the 130 and 180-day cut-offs periods.

	Sensitivity level	Relative False- Recency Rate	95% Lower bound	95% Upper bound
*BED+PwD*	75	28.3	13.8	47.9
*130-day*	80	35.0	17.0	48.3
*cut-off*	85	36.7	19.4	52.1
	90	40.0	23.2	68.5
*BED+PwD*	75	28.9	11.8	44.6
*180-day*	80	31.1	13.8	46.2
*cut-off*	85	31.1	18.1	53.2
	90	42.2	21.1	62.8
*LAg+PwD*	75	44.0	25.0	68.2
*130-day*	80	48.0	28.6	68.4
*cut-off*	85	48.0	27.5	73.3
	90	48.0	30.9	87.5
*LAg+PwD*	75	42.1	15.8	71.8
*180-day*	80	42.1	17.4	73.3
*cut-off*	85	42.1	18.5	71.8
	90	47.4	22.2	83.3

The table shows the reduction in the relative false-recency rate (rFRR) of the BED and LAg assays due to the PwD assay. A BED = 0.8 or LAg = 1.5 threshold was first used to screen the specimens for HIV infection recency. Specimens classified as recent were then re-screened using the PwD assay in order to reduce the rFRR while maintaining a 75%, 80%, 85% or 90% true-recency rate (sensitivity) of the BED or LAg assay. Since we are interested in the reduction of the rFRR by the PwD assay, we subtract this estimate from 100%. The results can be interpreted as follows: for the 180-day cut-off, the PwD assay reduces the rFRR by (100–42.2 =) 57.8% while maintaining a BED sensitivity of 90%. The table also gives the 95% confidence bounds for the reduction in the rFRR. The same result can be interpreted as follows: for the 180-day cut-off, the PwD assay reduces the rFRR by *at least* (100–62.8 =) 37.2% while maintaining a BED sensitivity of 90%.

Results show that the PwD assay reduces the rFRR by (100–42.2) 57.8%, or that it reduces the rFRR by *at least* (100–62.8) 37.2%, while maintaining a 90% sensitivity of the BED assay. Panel D of S2 Fig provides a graphical illustration of the reduction in the rFRR due to the BED plus PwD combination screening. The panel shows the rFRR estimate (red dot) on the ROC graph that corresponds with a 90% sensitivity (y-axis) and a 42.2% false-recent (x-axis) value. The red bar represents the 95% CI of the rFRR. Panels A-C of S2 Fig show that rFRR estimates at a sensitivity levels of 75%, 80% or 85% respectively, the values of which can be obtained from Table 3.

We further provide a data flow diagram in S3 Fig to demonstrate the procedure used to produce the results for Table 3. There were 217 time points that had values for *both* the PwD and BED assays, of which 134 were known to be recent. We first used a recommended BED threshold of ≤0.8 to classify 168 time points as recent infections. We then used a PwD threshold of ≤0.005 to re-screen these 168 time points in order to improve predictive accuracy. S3 Fig shows a reduction in the number of false-recent infections from 45 to 16 (64%) due to the PwD screening, while maintaining a BED sensitivity of 91.6%. This result differs slightly from that of Table 3, which is interpreted at an exact sensitivity of 90%.

**Fig 2:**
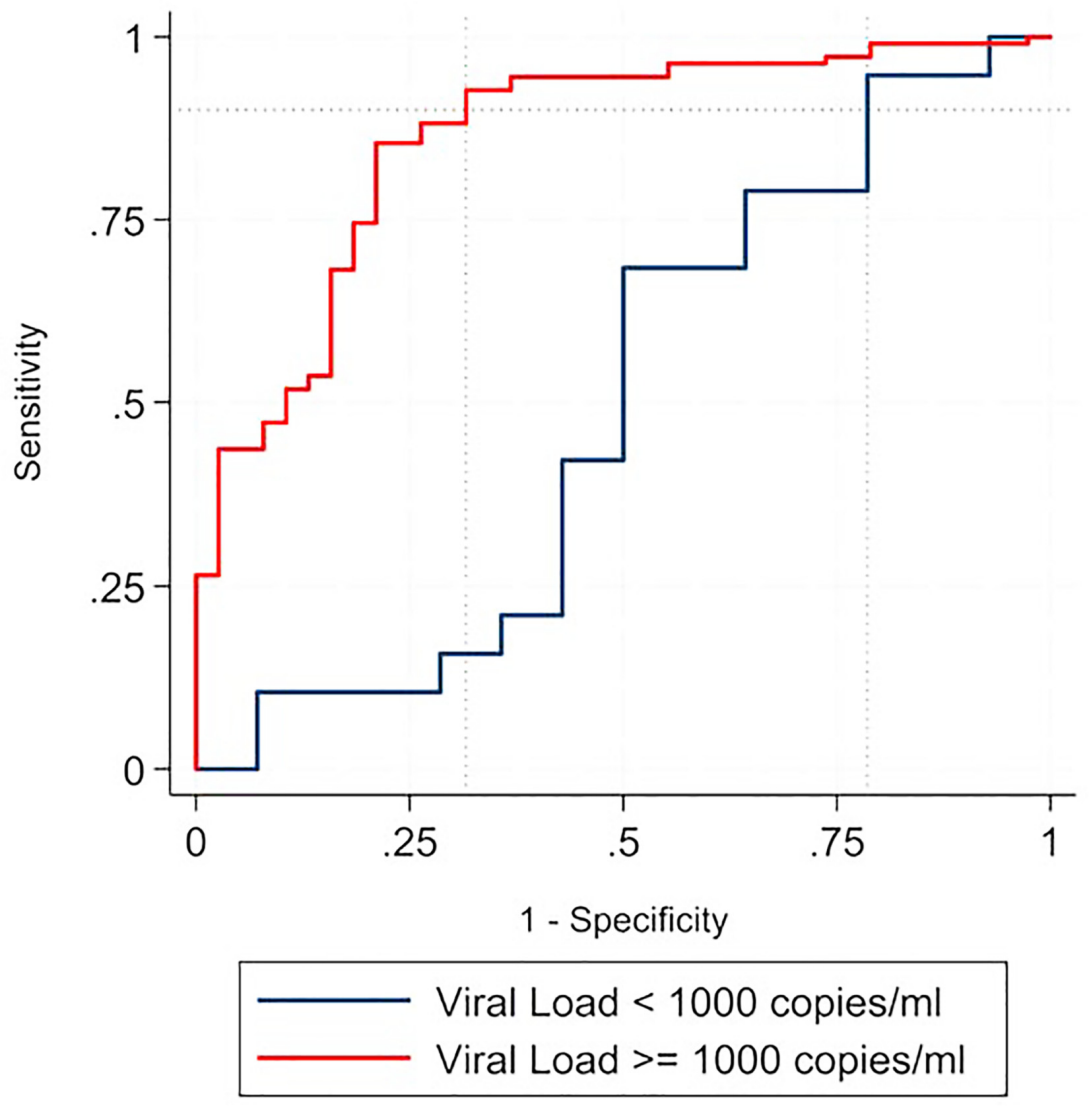
Shows a reduction in the relative false-recency rate (rFRR) when viral load information is added to the combination BED plus PwD screening procedure. The figure shows how additional biomarker information can be used to improve the combination screening procedure for the 180-day cut-off. We hypothesize that treatment naïve participants with viral loads ≤1000 copies/mL are more likely to be recently infected with HIV. Results show an rFRR estimate of 31.6% (95% CI: 11–63.1) at a 90% sensitivity level. Since we are interested in the reduction of the rFRR by the PwD assay, we subtract this estimate from 100%. Thus, the PwD assay reduces the rFRR by 68.4% (or by at least 36.9% given the upper bound of the 95% CI) while maintaining a BED sensitivity of 90% for the subsample of VL >1000 copies/mL specimens. The figure displays both ROC curves for the viral load covariate and the corresponding rFRR estimates (displayed by the dotted vertical lines).

**Fig 3:**
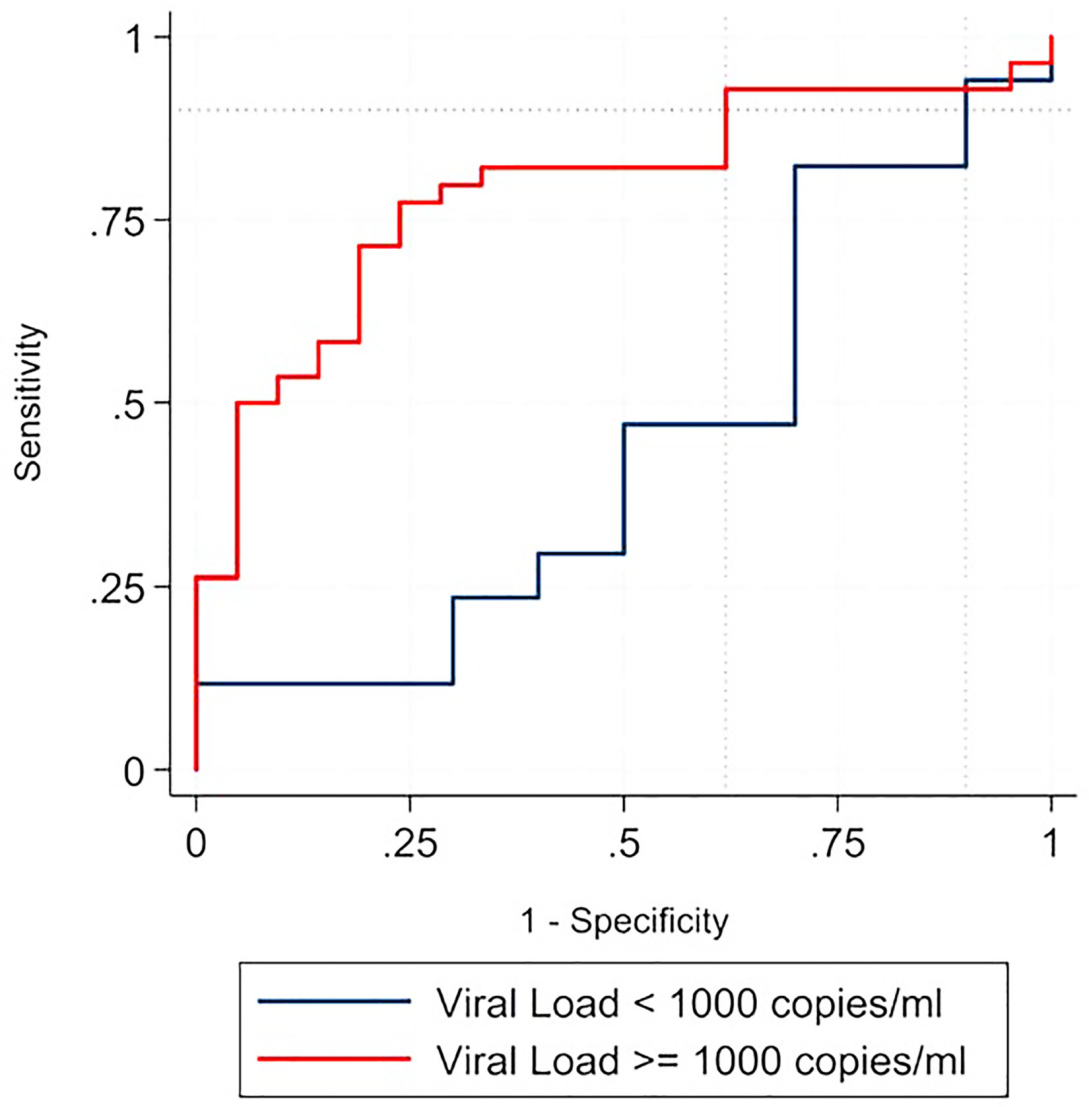
Shows a reduction in the relative false-recency rate when viral load information is added to the combination LAg plus PwD screening. The figure shows how additional biomarker information can be used to improve the combination screening procedure for the 130-day cut-off. We hypothesize that treatment naïve participants with viral loads ≤1000 copies/mL are more likely to be recently infected with HIV. Results show an rFRR estimate of 38.1% (95% CI: 15.8–88.6) at a 90% sensitivity level. Since we are interested in the reduction of the rFRR by the PwD assay, we subtract this estimate from 100%. Thus, the PwD assay reduces the rFRR by 61.9% (or by at least 11.4% given an upper bound of the 95% CI) while maintaining a LAg sensitivity of 90% for the subsample of VL <1000 copies/mL specimens. The figure displays both ROC curves for the viral load covariate and the corresponding rFRR estimates (displayed by the dotted vertical lines).

We then show how additional biomarker information can be used to improve the combination screening procedure. Here we hypothesize that treatment naïve participants with viral loads <1000 copies/mL are less likely to be recently infected with HIV. Fig 2 shows the BED plus PwD screening for the 180-day cut-off. The rFRR estimate is 31.6% (95% CI: 11.0–63.1), which shows that the PwD assay and VL information reduces rFRR by 68.4% (or by at least 36.9%) while maintaining a BED sensitivity of 90%. We also show this result for the LAg plus PwD combination screening for the 130-day cut-off in Fig 3.

Finally, we estimated MDRI’s for PwD using a threshold of 0.005, BED and LAg using standard thresholds of 0.8 and 1.5 respectively. PwD had an estimated MDRI of 128 days (95% CI 92–185). BED and LAg had estimated MDRIs of 267 days (95% 212–335) and 129 days (81–190), respectively (S4 Table)

## 4.0 Discussion and Conclusion

There is an urgent need in HIV research to classify infection recency using accurate, practical and cost effective methods [29, 78–81]. In this study, we evaluate the accuracy of a viral-based assay, HIV pairwise diversity (PwD), to identify participants recently infected with HIV. Our study provides information on the best-performing thresholds for the PwD assay, and compares this assay with two serologic-based assays, BED and LAg. We found that PwD threshold values in the range of 0.005 and 0.006 gave a high sensitivity and specificity for the 130 and 180-day cut-offs. These values are biologically feasible and consistent with previous work. For example, studies have determined that the mean pairwise sequence diversity of the HIV-1 *env* gene region increases at an approximately constant rate of 0.01 per year during early HIV infection [46]. Other studies using a different measure of HIV diversity, namely proportion of ambiguous sites, found that a threshold ranging from 0.0045 to 0.005 gave a high sensitivity for the 180-day cut-off [11]. Xia et. al. [82] show that a 0.006 diversity cut-off distinguished recent infections with both single and multiple infections.

The results of our study show that the PwD assay can accurately identify recent HIV infections. The PwD assay gave the best performance for the 130, 180, 360-day cut-offs according to the AUC estimates. PwD thresholds of 0.004 and 0.005 correctly classified a higher proportion of specimens when compared with BED and LAg thresholds of 0.8 and 1.5 respectively. We also evaluated a multi-assay algorithm (BED plus PwD or LAg plus PwD) to identify HIV infection recency. Our algorithm first uses an affordable, serologic based assay (BED or LAg) to identify a high proportion of true-recent HIV infections, and then the more sensitive PwD assay to reduce the percentage of specimens misclassified as recent infections. Combination screening significantly improved the classification of HIV infection recency. We found that the PwD assay was able to reduce the relative false-recency rate (rFRR) by approximately 52% while maintaining a LAg sensitivity of 90% for the 130-day cut-off. PwD reduced the rFRR by approximately 58% while maintaining a BED sensitivity of 90% for the 180-day cut-off. Results also show an improvement in accuracy when including biomarker information such as participant viral load (VL).

Prior research has shown that the presence or active use of ART can reduce HIV diversity and result in the misclassification of infection recency [62, 83]. The sensitivity of incidence assays can be maintained if auxiliary patient information on ART usage is collected at the same time as the blood specimen. The collection of additional information, such as VL or CD4 counts, has also been shown to improve the performance of bio-marker based assays to detect infection recency [12, 13, 32]. Our study confirms that the inclusion of VL as a covariate in the analysis significantly reduced the false-recent rate of the BED or LAg assays while maintaining a high sensitivity [12, 84, 85]. Collecting VL or CD4 count information may however increase operational costs. Some of these markers may not be readily available during routine cross-sectional surveys or for previously collected specimens. The PwD MDRI estimates are similar to LAg MDRI recently published [34, 86], although larger sample sets could help to evaluate different thresholds of PwD.

In this paper, we excluded time points with evidence of multiple founder variants or super-infection. Previous research has shown that multiplicity of infection can result in highly variable pairwise distances. PwD values calculated from multi-infection time points are likely to fall outside of the expected range, and do not give an accurate estimate of HIV diversity [61, 87]. Methods to better identify multi-infections in cross sectional sampling are currently being developed. The PwD assay may be of limited use in men who have sex with men (MSM) [88, 89] due to a high multiplicity of infection. However, more than 80% of all heterosexual HIV infections are seeded by a single founder strain [42–45], which is the main route of transmission in Botswana.

One current limitation associated with the wide-scale use of the PwD assay is the cost of genomic sequencing, which requires expensive laboratory equipment, the training of staff and the technically demanding task of generating single genomes or clonal sequences. The current cost of generating quasispecies from a single time point ranges from $150–200$ compared to $5.29 and $2.35 per test for LAg and BED, respectively. Nevertheless, we argue that the data generated from genome sequencing can address a range of research questions related to the timing of infections in transmission clusters, the number of strains infecting individuals, tropism of the virus and the selection of optimal drug regimens. In this regard, the costs of genome sequencing would be absorbed into a body of research initiatives and questions, rather than used exclusively for the generation of a viral diversity measure. It is also likely that expensive viral-based assays will become a moot point in the near future as the cost of genomics technology continues to decline.

In conclusion, serologic assays and their algorithms have become increasingly popular in recent years because they are based on antibody laboratory tests that are cheaper, quicker and relatively straightforward to implement at the population level [30, 31, 80, 90]. In this study, we show that a measure of HIV diversity can accurately classify infection recency. Our results show that BED plus PwD or LAg plus PwD combination screening has the potential to correctly identify a high proportion of recent HIV infections in a cost-effective manner. The use of bio-marker based assays and cross-sectional data to identify HIV infection recency presents a promising alternative to the resource-intensive approach of a longitudinal cohort design. With continued development, these assays hold the potential to accurately estimate HIV incidence, monitor the spread of the epidemic, evaluate the impact of treatment interventions and inform the design of vaccine and prevention trials.

## Supporting Information

S1 Fig Data flow diagram showing total time points and participants included in the final analysis. (PNG)

S2 Fig ROC graphs showing a reduction in the relative false-recency rate (rFRR) of the BED assay by the PwD assay for the <180-day cut-off. The figure gives an example of the reduction in the relative false-recency rate (rFRR) of the BED assay by the PwD assay for the 180-day cut-off. The panels A-D show the ROC curves for the four sensitivity levels. The y-axis is the sensitivity and the x-axis the false-recency rate (1 –specificity); the red point on each graph is the rFRR estimate along with its 95% CI, as shown by the red error bar. The PwD assay reduces the rFRR by 57.8% while maintaining a 90% sensitivity of the BED assay. The ROC graphs show that after performing combination screening, an rFRR estimate can be obtained for any sensitivity value between 0 and 1.0.
(PNG)

S3 Fig Flow chart of the combination BED plus PwD screening to identify HIV infection recency for the 180-day cut-off. Flow chart showing how the PwD assay can be combined with the BED assay to reduce the likelihood of a false-recent result (i.e., established infections misclassified as recent infections). A recommended BED assay threshold value of 0.8 was used to classify infection recency for the N = 217 specimens. This first screening correctly identified 123 of the 134 recent infections for the 180-day cut-off (true positives), giving a sensitivity of 91.8%. However, 45 of the 83 (54.2%) established specimens were falsely classified as recent. A PwD threshold of 0.005 was then used to screen the subset of specimens classified as recent (n = 168) by the BED assay. Results show that the secondary PwD screening reduces the false-recent infections by 64% (45 to 16 specimens) at a BED sensitivity of 87.8%. (This result differs slightly from that of Table 3, which is interpreted at an exact sensitivity of 90%.)
(PNG)

S4 Fig Distribution of time-points for the BED, LAg and PwD assays. The figure gives the analysed time points of sampling and sequencing in the study since the known time of seroconversion. Time in days post-seroconversion is shown on the x-axis.
(PNG)

S5 Fig Spaghetti plots for the BED, LAg and PwD time-points. (PNG)

S1 Table Performance of PwD threshold values to determine HIV Infection Recency for 130, 180, and 360-day cut-offs. The table shows the performance of the PwD threshold values to identify HIV infection recency. The range of values were selected according to rate of increase in the pairwise sequence diversity of the HIV-1 *env* gene region, which is approximately a constant rate of 0.01 per year during early infection. For example, a 180-day cut-off corresponds with a PwD value of 0.005. We selected thresholds values in the range of these biological values for each of the cut-off periods. For each threshold we obtained the sensitivity, specificity, their 95% CI, likelihood ratio, and percentage correctly classified. For the 130-day cut-off, a PwD threshold of 0.005 correctly identified 79.37% (95% CI: 62.83–95.9) of the recent infections (sensitivity) and correctly identified 72.57% (95% CI: 61.87–83.26) of the established infections (specificity), giving a percentage correctly classified of 76.15%.
(DOCX)

S2 Table Accession numbers for the reference sequences used. (DOCX)

S3 Table Area under the curve (AUC) for the PwD, BED, and LAg assays for shared time-points (n = 238). Table shows the results for the area under the curve (AUC) of a receiver operating characteristics (ROC) graph for the <130, <180- and <360-day cut-offs. Using only shared time-points (n = 238) significantly reduces the sample size and therefore the performance of the three assays. The performance of the three assays are therefore indistinguishable given the overlap in the confidence intervals of the AUC estimates.
(DOCX)

S4 Table Mean Duration of Recent Infection for BED, LAg and PwD Assays. Table shows the estimated Mean Duration of Recent Infection (MDRI), average time ‘recent’ while infected for less than some time cut-off T for the BED, LAg and PwD assays.
(DOCX)

## Abbreviations

PwD: pairwise diversity
BED: Calypte Incidence Assay
LAg: Limiting Antigen Assay
ROC: receiver operator characteristics
FRR: false-recency rate
ART: antiretroviral treatment
TPR: true positive rate
AUC: area under the curve
