# Supplementary figures and images for: Analysis of Viral Diversity in Relation to the Recency of HIV-1C Infection in Botswana
Source: PLoS One. 2016 Aug 23;11(8):e0160649. doi: 10.1371/journal.pone.0160649 (PMC4994946; doi:10.1371/journal.pone.0160649)

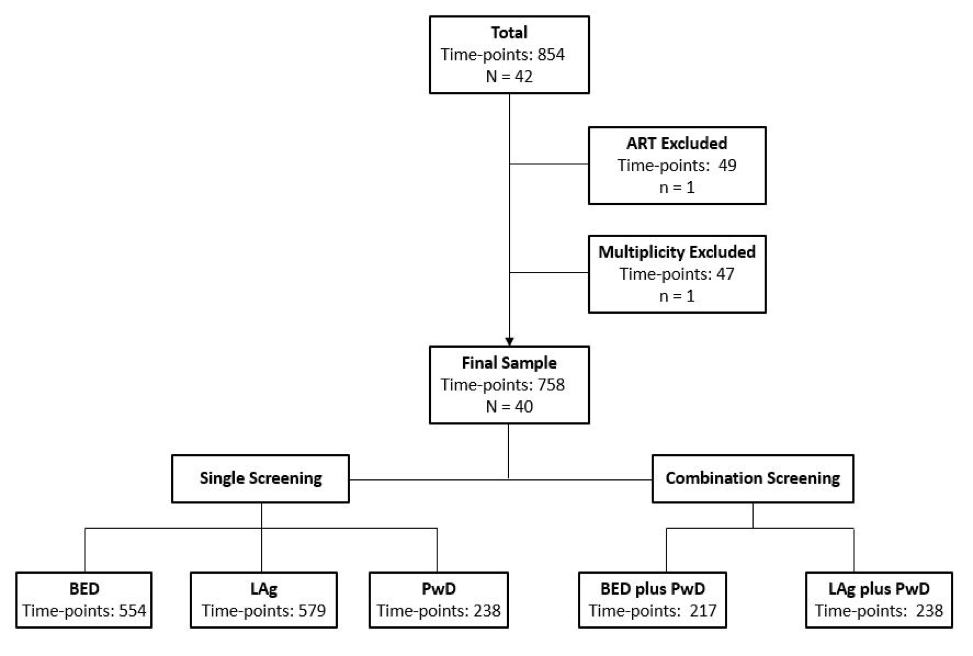

Supplement: S1 Fig — (PNG) [file pone.0160649.s001.png]

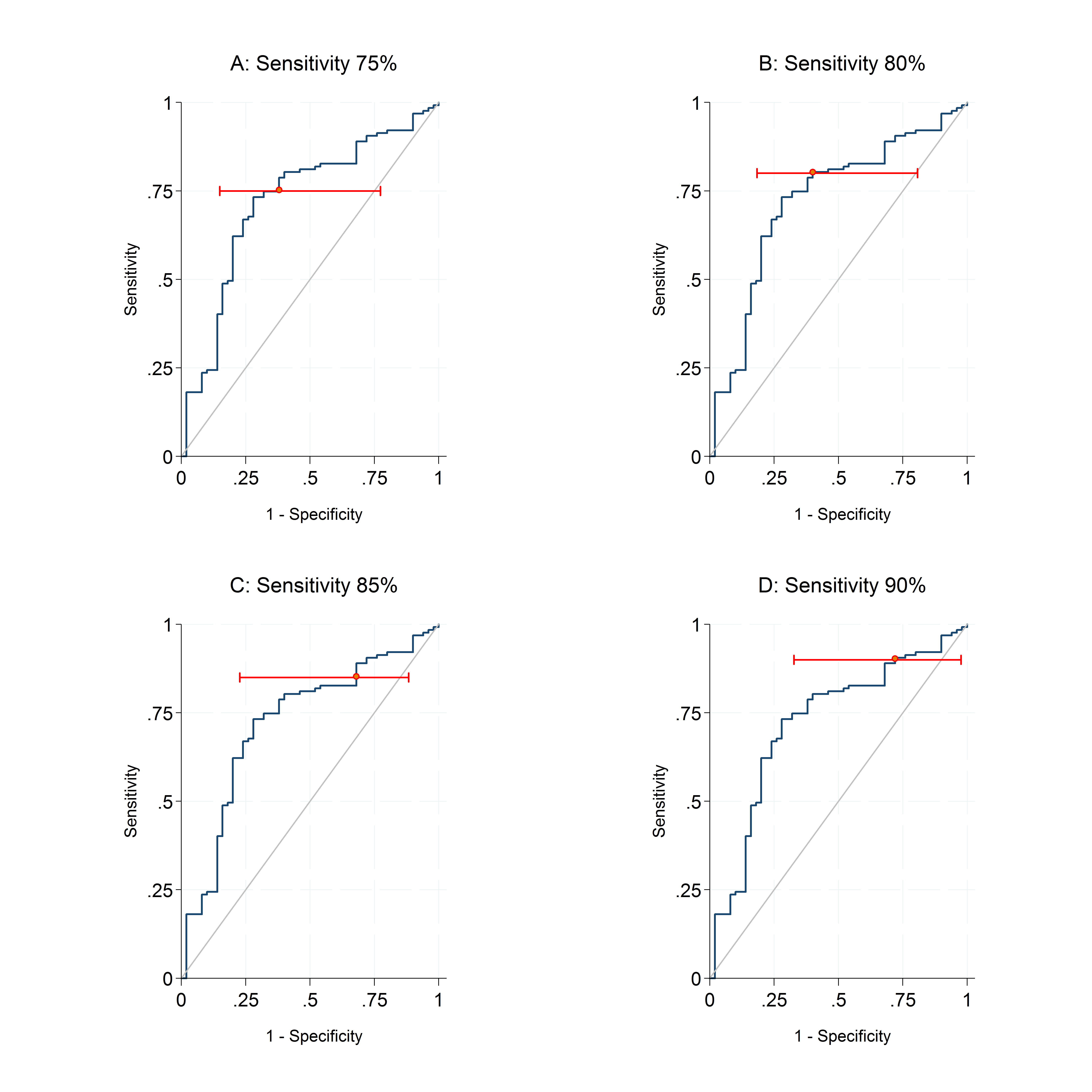

Supplement: S2 Fig — The figure gives an example of the reduction in the relative false-recency rate (rFRR) of the BED assay by the PwD assay for the 180-day cut-off. The panels A-D show the ROC curves for the four sensitivity levels. The y-axis is the sensitivity and the x-axis the false-recency rate (1 –specificity); the red point on each graph is the rFRR estimate along with its 95% CI, as shown by the red error bar. The PwD assay reduces the rFRR by 57.8% while maintaining a 90% sensitivity of the BED assay. The ROC graphs show that after performing combination screening, an rFRR estimate can be obtained for any sensitivity value between 0 and 1.0. (PNG) [file pone.0160649.s002.png]

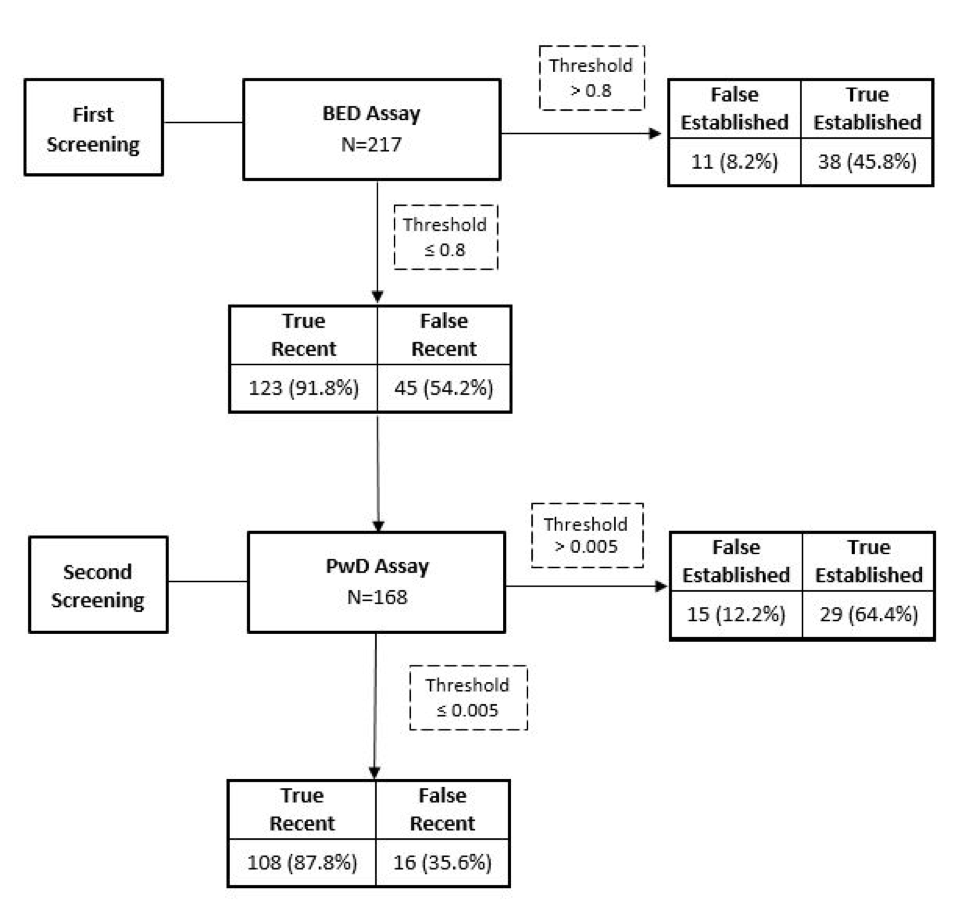

Supplement: S3 Fig — Flow chart showing how the PwD assay can be combined with the BED assay to reduce the likelihood of a false-recent result (i.e., established infections misclassified as recent infections). A recommended BED assay threshold value of 0.8 was used to classify infection recency for the N = 217 specimens. This first screening correctly identified 123 of the 134 recent infections for the 180-day cut-off (true positives), giving a sensitivity of 91.8%. However, 45 of the 83 (54.2%) established specimens were falsely classified as recent. A PwD threshold of 0.005 was then used to screen the subset of specimens classified as recent (n = 168) by the BED assay. Results show that the secondary PwD screening reduces the false-recent infections by 64% (45 to 16 specimens) at a BED sensitivity of 87.8%. (This result differs slightly from that of Table 3, which is interpreted at an exact sensitivity of 90%.) (PNG) [file pone.0160649.s003.png]

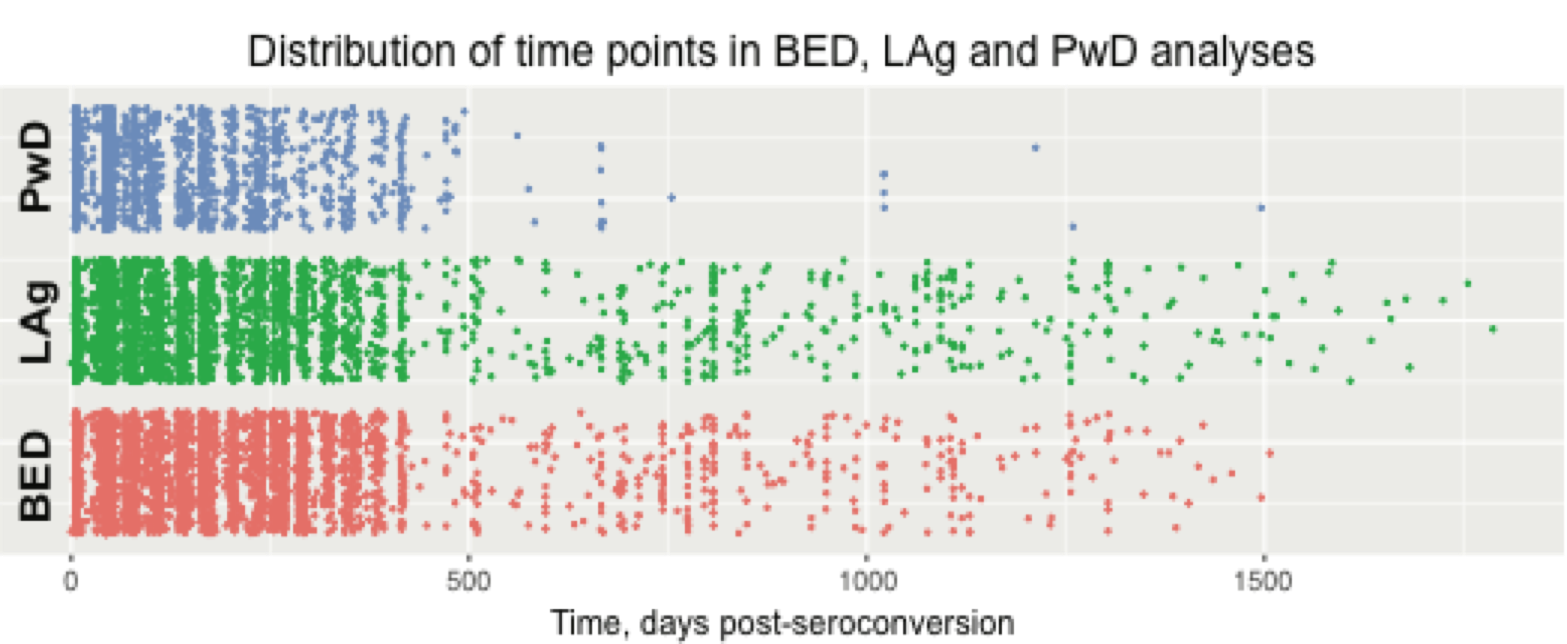

Supplement: S4 Fig — The figure gives the analysed time points of sampling and sequencing in the study since the known time of seroconversion. Time in days post-seroconversion is shown on the x-axis. (PNG) [file pone.0160649.s004.png]

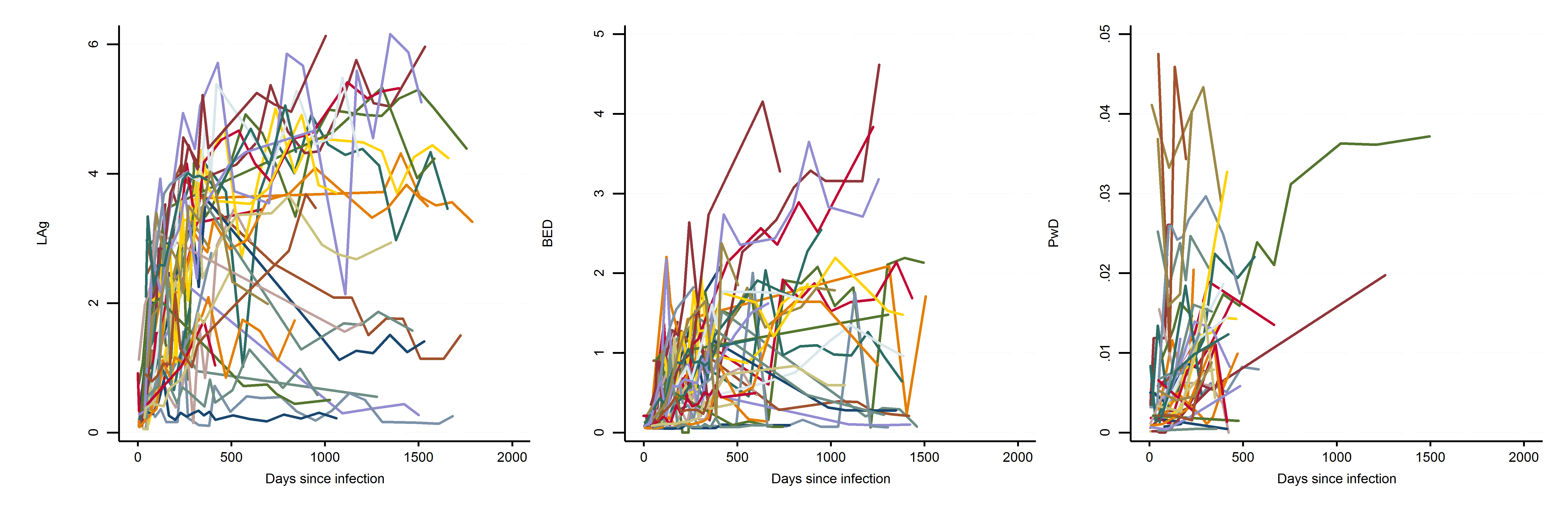

Supplement: S5 Fig — (PNG) [file pone.0160649.s005.png]
