# Supplementary material for: Analysis of Viral Diversity in Relation to the Recency of HIV-1C Infection in Botswana
Source: PLoS One. 2016 Aug 23;11(8):e0160649. doi: 10.1371/journal.pone.0160649 (PMC4994946; doi:10.1371/journal.pone.0160649)
Supplement: S1 Table — The table shows the performance of the PwD threshold values to identify HIV infection recency. The range of values were selected according to rate of increase in the pairwise sequence diversity of the HIV-1 env gene region, which is approximately a constant rate of 0.01 per year during early infection. For example, a 180-day cut-off corresponds with a PwD value of 0.005. We selected thresholds values in the range of these biological values for each of the cut-off periods. For each threshold we obtained the sensitivity, specificity, their 95% CI, likelihood ratio, and percentage correctly classified. For the 130-day cut-off, a PwD threshold of 0.005 correctly identified 79.37% (95% CI: 62.83–95.9) of the recent infections (sensitivity) and correctly identified 72.57% (95% CI: 61.87–83.26) of the established infections (specificity), giving a percentage correctly classified of 76.15%. (DOCX) [file pone.0160649.s006.docx]

Table S1: Performance of PwD threshold values to determine HIV Infection Recency for 130, 180, and 360-day cut-offs.

| Threshold | Sensitivity | 95% LB | 95% UB | Specificity | 95% LB | 95% UB | % Correctly Classified |
| --- | --- | --- | --- | --- | --- | --- | --- |
| *130-day cut-off* |  |  |  |  |  |  |  |
| 0.0005 | 19.84 | 0.00 | 41.37 | 96.46 | 92.19 | 100.00 | 56.07 |
| 0.0010 | 23.02 | 1.51 | 44.52 | 96.46 | 92.19 | 100.00 | 57.74 |
| 0.0015 | 27.78 | 6.77 | 48.79 | 92.92 | 87.48 | 98.36 | 58.58 |
| 0.0020 | 44.44 | 21.69 | 67.20 | 91.15 | 84.91 | 97.39 | 66.53 |
| 0.0025 | 48.41 | 26.02 | 70.81 | 87.61 | 79.76 | 95.46 | 66.95 |
| 0.0030 | 55.56 | 34.74 | 76.37 | 84.07 | 75.64 | 92.50 | 69.04 |
| 0.0035 | 72.22 | 54.01 | 90.43 | 82.30 | 73.83 | 90.77 | 76.99 |
| 0.0040 | 76.19 | 59.31 | 93.07 | 79.65 | 70.45 | 88.84 | 77.82 |
| 0.0045 | 76.98 | 60.16 | 93.81 | 76.11 | 66.68 | 85.54 | 76.57 |
| 0.0050 | 79.37 | 62.83 | 95.90 | 72.57 | 61.87 | 83.26 | 76.15 |
| 0.0055 | 88.89 | 81.57 | 96.21 | 69.03 | 57.55 | 80.50 | 79.50 |
| 0.0060 | 90.48 | 83.79 | 97.16 | 67.26 | 55.21 | 79.30 | 79.50 |
| 0.0065 | 90.48 | 83.79 | 97.16 | 63.72 | 49.80 | 77.63 | 77.82 |
| 0.0070 | 92.06 | 86.00 | 98.13 | 61.95 | 47.35 | 76.54 | 77.82 |
| 0.0075 | 93.65 | 87.84 | 99.47 | 58.41 | 43.76 | 73.05 | 76.99 |
| 0.0080 | 93.65 | 87.84 | 99.47 | 50.44 | 35.78 | 65.11 | 73.22 |
| *180-day cut-off* |  |  |  |  |  |  |  |
| 0.0010 | 20.00 | 0.97 | 39.03 | 95.74 | 90.60 | 100.00 | 49.79 |
| 0.0015 | 24.83 | 6.09 | 43.57 | 92.55 | 86.37 | 98.74 | 51.46 |
| 0.0020 | 39.31 | 18.64 | 59.98 | 90.43 | 83.14 | 97.71 | 59.41 |
| 0.0025 | 44.14 | 23.94 | 64.34 | 88.30 | 79.54 | 97.05 | 61.51 |
| 0.0030 | 51.03 | 32.35 | 69.72 | 85.11 | 75.62 | 94.59 | 64.44 |
| 0.0035 | 66.21 | 49.36 | 83.05 | 84.04 | 74.54 | 93.54 | 73.22 |
| 0.0040 | 69.66 | 53.92 | 85.39 | 80.85 | 70.60 | 91.10 | 74.06 |
| 0.0045 | 71.72 | 56.25 | 87.20 | 78.72 | 68.25 | 89.20 | 74.48 |
| 0.0050 | 74.48 | 59.16 | 89.80 | 75.53 | 64.52 | 86.54 | 74.90 |
| 0.0055 | 82.76 | 73.25 | 92.26 | 71.28 | 59.24 | 83.31 | 78.24 |
| 0.0060 | 84.14 | 75.38 | 92.90 | 69.15 | 56.15 | 82.15 | 78.24 |
| 0.0065 | 84.83 | 76.46 | 93.20 | 65.96 | 51.34 | 80.58 | 77.41 |
| 0.0070 | 86.90 | 79.31 | 94.48 | 64.89 | 49.57 | 80.22 | 78.24 |
| 0.0075 | 89.66 | 82.90 | 96.41 | 62.77 | 47.10 | 78.43 | 79.08 |
| 0.0080 | 92.41 | 86.44 | 98.39 | 57.45 | 40.97 | 73.92 | 78.66 |
| 0.0085 | 93.10 | 87.30 | 98.91 | 55.32 | 38.31 | 72.33 | 78.24 |
| 0.0090 | 93.79 | 88.19 | 99.40 | 48.94 | 32.88 | 65.00 | 76.15 |
| *360-day cut-off* |  |  |  |  |  |  |  |
| 0.0040 | 55.07 | 41.36 | 68.78 | 84.38 | 67.37 | 100.00 | 59.00 |
| 0.0045 | 57.49 | 44.17 | 70.81 | 84.38 | 67.37 | 100.00 | 61.09 |
| 0.0050 | 60.39 | 47.40 | 73.37 | 81.25 | 63.22 | 99.28 | 63.18 |
| 0.0055 | 67.63 | 56.97 | 78.30 | 78.13 | 59.64 | 96.61 | 69.04 |
| 0.0060 | 69.08 | 59.17 | 78.99 | 75.00 | 55.23 | 94.77 | 69.87 |
| 0.0065 | 71.01 | 61.15 | 80.88 | 75.00 | 55.23 | 94.77 | 71.55 |
| 0.0070 | 72.46 | 63.09 | 81.84 | 71.88 | 50.82 | 92.93 | 72.38 |
| 0.0075 | 74.88 | 66.14 | 83.62 | 68.75 | 46.43 | 91.07 | 74.06 |
| 0.0080 | 78.74 | 70.26 | 87.22 | 65.63 | 41.52 | 89.73 | 76.99 |
| 0.0085 | 79.23 | 70.87 | 87.59 | 59.38 | 31.26 | 87.49 | 76.57 |
| 0.0090 | 82.61 | 74.80 | 90.41 | 59.38 | 31.26 | 87.49 | 79.50 |
| 0.0095 | 83.57 | 76.25 | 90.90 | 59.38 | 31.26 | 87.49 | 80.33 |
| 0.0100 | 84.06 | 76.80 | 91.31 | 56.25 | 26.39 | 86.11 | 80.33 |
| 0.0105 | 84.54 | 77.45 | 91.64 | 56.25 | 26.39 | 86.11 | 80.75 |
| 0.0110 | 85.51 | 78.46 | 92.56 | 56.25 | 26.39 | 86.11 | 81.59 |
| 0.0115 | 86.96 | 80.08 | 93.83 | 56.25 | 26.39 | 86.11 | 82.85 |
| 0.0120 | 88.89 | 82.50 | 95.28 | 56.25 | 26.39 | 86.11 | 84.52 |
| 0.0125 | 89.86 | 83.76 | 95.95 | 53.13 | 22.31 | 83.94 | 84.94 |
| 0.0130 | 91.79 | 86.65 | 96.92 | 53.13 | 22.31 | 83.94 | 86.61 |

The table shows the performance of the PwD threshold values to identify HIV infection recency. The range of values were selected according to rate of increase in the pairwise sequence diversity of the HIV-1 *env* gene region, which is approximately a constant rate of 0.01 per year during early infection. For example, a 180-day cut-off corresponds with a PwD value of 0.005. We selected thresholds values in the range of these biological values for each of the cut-off periods. For each threshold we obtained the sensitivity, specificity, their 95% CI, likelihood ratio, and percentage correctly classified. For the 130-day cut-off, a PwD threshold of 0.005 correctly identified 79.37% (95% CI: 62.83-95.9) of the recent infections (sensitivity) and correctly identified 72.57% (95% CI: 61.87-83.26) of the established infections (specificity), giving a percentage correctly classified of 76.15%.
