# Supplementary material for: Analysis of Viral Diversity in Relation to the Recency of HIV-1C Infection in Botswana
Source: PLoS One. 2016 Aug 23;11(8):e0160649. doi: 10.1371/journal.pone.0160649 (PMC4994946; doi:10.1371/journal.pone.0160649)
Supplement: S2 Table — (DOCX) [file pone.0160649.s007.docx]

Table S2: Accession numbers for the reference sequences used

| AB097871 | AF443086 | AY463222 | AY529679 | AY901973 | DQ093597 | DQ351218 | DQ382372 | DQ445633 | EU863417 |
| --- | --- | --- | --- | --- | --- | --- | --- | --- | --- |
| AB254141 | AF443087 | AY463223 | AY585264 | AY901974 | DQ093598 | DQ351219 | DQ382373 | DQ445634 | EU863418 |
| AB254142 | AF443088 | AY463224 | AY585265 | AY901975 | DQ093599 | DQ351220 | DQ382374 | DQ445635 | EU863419 |
| AB254143 | AF443089 | AY463225 | AY585266 | AY901976 | DQ093600 | DQ351221 | DQ382375 | DQ445637 | EU863420 |
| AB254146 | AF443090 | AY463226 | AY585267 | AY901977 | DQ093601 | DQ351222 | DQ382376 | DQ447266 | EU863421 |
| AB254148 | AF443091 | AY463227 | AY585268 | AY901978 | DQ093602 | DQ351223 | DQ382377 | DQ447267 | EU863422 |
| AB254149 | AF443092 | AY463228 | AY669739 | AY901979 | DQ093604 | DQ351224 | DQ382378 | DQ447268 | EU863423 |
| AB254150 | AF443093 | AY463229 | AY669749 | AY901980 | DQ093605 | DQ351225 | DQ382379 | DQ447269 | EU863424 |
| AB254155 | AF443094 | AY463230 | AY703908 | AY901981 | DQ093607 | DQ351226 | DQ382380 | DQ447270 | EU863425 |
| AB254156 | AF443095 | AY463231 | AY703909 | DQ011165 | DQ164104 | DQ351227 | DQ388514 | DQ447272 | EU863426 |
| AB485645 | AF443097 | AY463232 | AY703910 | DQ011166 | DQ164106 | DQ351228 | DQ388515 | DQ499756 | EU863427 |
| AF095831 | AF443098 | AY463233 | AY703911 | DQ011167 | DQ164107 | DQ351229 | DQ388516 | DQ499757 | EU863428 |
| AF110959 | AF443099 | AY463234 | AY713413 | DQ011169 | DQ164108 | DQ351230 | DQ388517 | DQ499760 | EU863429 |
| AF110962 | AF443100 | AY463235 | AY772690 | DQ011170 | DQ164109 | DQ351231 | DQ396364 | DQ499762 | EU863430 |
| AF110967 | AF443101 | AY463236 | AY772691 | DQ011171 | DQ164110 | DQ351232 | DQ396365 | DQ499765 | EU863431 |
| AF110969 | AF443102 | AY463237 | AY772692 | DQ011172 | DQ164111 | DQ351234 | DQ396367 | DQ499766 | EU863432 |
| AF110972 | AF443103 | AY494971 | AY772693 | DQ011173 | DQ164113 | DQ351235 | DQ396368 | DQ499768 | EU863433 |
| AF110973 | AF443104 | AY522721 | AY772694 | DQ011174 | DQ164114 | DQ351237 | DQ396369 | DQ499769 | EU863434 |
| AF110976 | AF443105 | AY522722 | AY772695 | DQ011175 | DQ164115 | DQ369976 | DQ396370 | DQ499770 | EU863435 |
| AF286224 | AF443107 | AY522723 | AY772696 | DQ011176 | DQ164117 | DQ369977 | DQ396371 | DQ499771 | EU863436 |
| AF286225 | AF443108 | AY522724 | AY772698 | DQ011177 | DQ164118 | DQ369978 | DQ396372 | DQ499772 | EU863437 |
| AF286227 | AF443109 | AY522725 | AY772699 | DQ011178 | DQ164119 | DQ369979 | DQ396373 | DQ499773 | EU863438 |
| AF290027 | AF443110 | AY522726 | AY772700 | DQ011179 | DQ164121 | DQ369980 | DQ396374 | DQ499774 | EU863439 |
| AF391230 | AF443111 | AY522727 | AY772701 | DQ011180 | DQ164122 | DQ369981 | DQ396375 | DQ499775 | EU863440 |
| AF391232 | AF443112 | AY522728 | AY805330 | DQ056404 | DQ164126 | DQ369982 | DQ396376 | DQ499776 | EU863441 |
| AF391233 | AF443113 | AY522729 | AY878054 | DQ056405 | DQ164127 | DQ369983 | DQ396377 | DQ904348 | EU863442 |
| AF391234 | AF443114 | AY522730 | AY878055 | DQ056406 | DQ164129 | DQ369984 | DQ396378 | EF203957 | EU863443 |
| AF391235 | AF443115 | AY522731 | AY878056 | DQ056408 | DQ275642 | DQ369985 | DQ396380 | EF203963 | EU863444 |
| AF391238 | AF544007 | AY522732 | AY878057 | DQ056409 | DQ275643 | DQ369986 | DQ396381 | EF203965 | EU863445 |
| AF391240 | AF544008 | AY522733 | AY878058 | DQ056410 | DQ275644 | DQ369987 | DQ396382 | EF203968 | EU863446 |
| AF391242 | AX457092 | AY522734 | AY878059 | DQ056411 | DQ275645 | DQ369988 | DQ396383 | EF203970 | EU863447 |
| AF391243 | AY043175 | AY522735 | AY878060 | DQ056412 | DQ275646 | DQ369989 | DQ396384 | EF203973 | EU863448 |
| AF391245 | AY043176 | AY522736 | AY878061 | DQ056413 | DQ275647 | DQ369990 | DQ396385 | EF203976 | EU863449 |
| AF391247 | AY118165 | AY529659 | AY878062 | DQ056414 | DQ275648 | DQ369991 | DQ396386 | EF203980 | EU863450 |
| AF391249 | AY118166 | AY529660 | AY878063 | DQ056415 | DQ275649 | DQ369992 | DQ396387 | EF203982 | EU863451 |
| AF391250 | AY158533 | AY529661 | AY878064 | DQ056416 | DQ275650 | DQ369993 | DQ396388 | EF203983 | FJ443159 |
| AF411966 | AY158534 | AY529662 | AY878065 | DQ056417 | DQ275651 | DQ369994 | DQ396389 | EF203985 | FJ443166 |
| AF411967 | AY158535 | AY529664 | AY878068 | DQ056418 | DQ275652 | DQ369995 | DQ396390 | EU166353 | FJ443177 |
| AF443074 | AY228556 | AY529665 | AY878069 | DQ093585 | DQ275653 | DQ369996 | DQ396391 | EU166379 | FJ443196 |
| AF443075 | AY228557 | AY529666 | AY878070 | DQ093586 | DQ275654 | DQ369997 | DQ396392 | EU166413 | FJ443209 |
| AF443076 | AY423971 | AY529667 | AY878071 | DQ093587 | DQ275655 | DQ382361 | DQ396393 | EU166439 | FJ443240 |
| AF443077 | AY423984 | AY529668 | AY878072 | DQ093588 | DQ275656 | DQ382362 | DQ396394 | EU166576 | FJ443253 |
| AF443078 | AY424079 | AY529669 | AY901965 | DQ093589 | DQ275657 | DQ382363 | DQ396395 | EU166653 | FJ443274 |
| AF443079 | AY424138 | AY529670 | AY901966 | DQ093590 | DQ275658 | DQ382364 | DQ396399 | EU166681 | FJ443367 |
| AF443080 | AY424163 | AY529672 | AY901967 | DQ093591 | DQ275659 | DQ382365 | DQ411853 | EU166718 | FJ443379 |
| AF443081 | AY463217 | AY529673 | AY901968 | DQ093592 | DQ275660 | DQ382366 | DQ422948 | EU166759 | FJ443455 |
| AF443082 | AY463218 | AY529675 | AY901969 | DQ093593 | DQ275661 | DQ382367 | DQ435682 | EU166779 | FJ443474 |
| AF443083 | AY463219 | AY529676 | AY901970 | DQ093594 | DQ275664 | DQ382369 | DQ435683 | EU293444 | FJ443520 |
| AF443084 | AY463220 | AY529677 | AY901971 | DQ093595 | DQ351216 | DQ382370 | DQ435684 | EU293450 | FJ443533 |
| AF443085 | AY463221 | AY529678 | AY901972 | DQ093596 | DQ351217 | DQ382371 | DQ445632 | EU863416 | FJ443548 |

| FJ443557 | FJ859428 | GU216803 | HM036960 | HM204617 | HM623592 | HQ595761 | HQ625576 | JF722826 | JN188292 |
| --- | --- | --- | --- | --- | --- | --- | --- | --- | --- |
| FJ443575 | FJ859592 | GU216837 | HM036983 | HM204618 | HM623593 | HQ595762 | HQ625577 | JF722835 | JN681219 |
| FJ443600 | GQ485312 | GU329048 | HM037006 | HM204619 | HM623594 | HQ595765 | HQ625578 | JF722849 | JN681220 |
| FJ443624 | GQ485415 | GU329053 | HM068596 | HM204622 | HM623595 | HQ595766 | HQ625579 | JF722862 | JN681221 |
| FJ443644 | GQ999973 | GU329063 | HM068598 | HM204624 | HM623596 | HQ595810 | HQ625580 | JF722867 | JN681222 |
| FJ443666 | GQ999977 | GU329078 | HM070449 | HM215360 | HM623597 | HQ595855 | HQ625581 | JF722874 | JN681227 |
| FJ443690 | GQ999985 | GU329094 | HM070491 | HM215361 | HM623598 | HQ595978 | HQ625582 | JF722886 | JN681229 |
| FJ443713 | GQ999987 | GU329107 | HM070529 | HM215362 | HM623599 | HQ596030 | HQ625583 | JF722892 | JN681232 |
| FJ443734 | GQ999990 | GU329131 | HM070571 | HM623548 | HM623600 | HQ596137 | HQ625584 | JF722898 | JN681233 |
| FJ443757 | GU080160 | GU329144 | HM070630 | HM623549 | HM623601 | HQ615941 | HQ625585 | JF722905 | JN681234 |
| FJ443782 | GU080161 | GU329154 | HM070674 | HM623550 | HM623602 | HQ615942 | HQ625586 | JF722919 | JN681236 |
| FJ443839 | GU080162 | GU329164 | HM070791 | HM623551 | HM623603 | HQ615943 | HQ625587 | JF722952 | JN681237 |
| FJ443861 | GU080163 | GU329184 | HM179745 | HM623552 | HM623604 | HQ615944 | HQ625588 | JF723001 | JN681238 |
| FJ443883 | GU080164 | GU329195 | HM179764 | HM623553 | HM623605 | HQ615945 | HQ625589 | JF723011 | JN681239 |
| FJ443906 | GU080165 | GU329205 | HM179793 | HM623554 | HM623606 | HQ615946 | HQ625590 | JF723021 | JN681240 |
| FJ443923 | GU080166 | GU329216 | HM179818 | HM623555 | HM623607 | HQ615947 | HQ625591 | JF723036 | JN681241 |
| FJ443938 | GU080167 | GU329236 | HM179844 | HM623556 | HM623608 | HQ615948 | HQ625592 | JF723048 | JN681242 |
| FJ443963 | GU080168 | GU329257 | HM179874 | HM623557 | HM623609 | HQ615949 | HQ625593 | JF723054 | JN681243 |
| FJ443989 | GU080169 | GU329271 | HM179900 | HM623558 | HM623610 | HQ615950 | HQ625594 | JF723061 | JN681244 |
| FJ444007 | GU080170 | GU329289 | HM179927 | HM623559 | HM623611 | HQ615951 | HQ625595 | JF723080 | JN681245 |
| FJ444035 | GU080171 | GU329300 | HM179956 | HM623561 | HM638616 | HQ615952 | HQ707833 | JF723099 | JN681246 |
| FJ444058 | GU080172 | GU329310 | HM204581 | HM623562 | HM638668 | HQ615953 | HQ707850 | JF723123 | JN681247 |
| FJ444077 | GU080173 | GU329321 | HM204583 | HM623563 | HM638722 | HQ615954 | HQ707867 | JF723137 | JN681248 |
| FJ444092 | GU080174 | GU329333 | HM204584 | HM623564 | HM638780 | HQ615955 | HQ707882 | JF723152 | JN681249 |
| FJ444120 | GU080175 | GU329354 | HM204585 | HM623565 | HM638826 | HQ615956 | HQ707897 | JF723168 | JN681250 |
| FJ444159 | GU080176 | GU329364 | HM204587 | HM623566 | HM638888 | HQ615957 | HQ707911 | JF723179 | JN681252 |
| FJ444186 | GU080177 | GU329375 | HM204588 | HM623567 | HM638963 | HQ615958 | HQ707925 | JN108036 | JN681253 |
| FJ444208 | GU080178 | GU329389 | HM204590 | HM623568 | HM639009 | HQ615959 | HQ707944 | JN108053 | JN681254 |
| FJ444230 | GU080179 | GU329399 | HM204591 | HM623569 | HM639073 | HQ615960 | HQ707963 | JN108112 | JN681255 |
| FJ444253 | GU080180 | GU329415 | HM204592 | HM623570 | HM639117 | HQ615961 | HQ707976 | JN108128 | JN681256 |
| FJ444281 | GU080181 | GU329437 | HM204594 | HM623571 | HM639160 | HQ615962 | HQ707990 | JN108153 | JN681257 |
| FJ444305 | GU080182 | GU329458 | HM204595 | HM623572 | HM639204 | HQ615963 | HQ708007 | JN108162 | JN681258 |
| FJ444325 | GU080183 | GU329481 | HM204597 | HM623573 | HQ143465 | HQ615964 | HQ708019 | JN108177 | JN681259 |
| FJ444347 | GU080184 | GU329490 | HM204598 | HM623574 | HQ595742 | HQ615965 | HQ708034 | JN108190 | JN687694 |
| FJ444369 | GU080185 | GU329500 | HM204599 | HM623575 | HQ595743 | HQ615966 | HQ708047 | JN108333 | JN687704 |
| FJ444392 | GU080186 | GU329510 | HM204600 | HM623576 | HQ595744 | HQ615967 | HQ708063 | JN108376 | JN687706 |
| FJ444416 | GU080187 | GU939049 | HM204601 | HM623577 | HQ595745 | HQ615982 | HQ708079 | JN108384 | JN687717 |
| FJ444436 | GU080188 | GU939062 | HM204602 | HM623578 | HQ595746 | HQ615983 | HQ708095 | JN108396 | JN687718 |
| FJ444457 | GU080189 | GU939124 | HM204604 | HM623579 | HQ595747 | HQ615984 | HQ708109 | JN108410 | JN687726 |
| FJ444480 | GU080190 | GU939129 | HM204605 | HM623580 | HQ595748 | HQ625565 | HQ708127 | JN108426 | JN687728 |
| FJ444501 | GU080191 | GU939143 | HM204606 | HM623581 | HQ595749 | HQ625566 | HQ708134 | JN108455 | JN687730 |
| FJ444523 | GU080192 | HM036739 | HM204607 | HM623582 | HQ595750 | HQ625567 | JF680908 | JN108469 | JN687731 |
| FJ444543 | GU080193 | HM036760 | HM204608 | HM623583 | HQ595751 | HQ625568 | JF722674 | JN108511 | JN687734 |
| FJ444566 | GU080194 | HM036792 | HM204609 | HM623584 | HQ595752 | HQ625569 | JF722677 | JN108549 | JN687735 |
| FJ444589 | GU080195 | HM036812 | HM204610 | HM623585 | HQ595753 | HQ625570 | JF722697 | JN108582 | JN687736 |
| FJ444611 | GU080196 | HM036832 | HM204611 | HM623586 | HQ595756 | HQ625571 | JF722719 | JN108600 | JN687821 |
| FJ846628 | GU080197 | HM036843 | HM204613 | HM623587 | HQ595757 | HQ625572 | JF722738 | JN108632 | JN977604 |
| FJ854750 | GU080198 | HM036864 | HM204614 | HM623588 | HQ595758 | HQ625573 | JF722767 | JN108670 | JN983803 |
| FJ854842 | GU080199 | HM036903 | HM204615 | HM623590 | HQ595759 | HQ625574 | JF722793 | JN108709 | JN983804 |
| FJ855076 | GU216724 | HM036933 | HM204616 | HM623591 | HQ595760 | HQ625575 | JF722819 | JN108746 | JN983805 |

| JQ061131 | KC154019 | KC247375 | KC894123 | KF725917 | KF725974 | KF770430 |  |
| --- | --- | --- | --- | --- | --- | --- | --- |
| JQ777026 | KC154020 | KC634109 | KC894128 | KF725919 | KF725975 | KJ700458 |  |
| JQ777046 | KC154021 | KC634136 | KC894130 | KF725920 | KF725976 | KP109494 |  |
| JQ777061 | KC154022 | KC634164 | KC894132 | KF725921 | KF725978 | KP109495 |  |
| JQ777087 | KC154023 | KC634184 | KC894383 | KF725922 | KF725979 | KP109496 |  |
| JQ777111 | KC154024 | KC862610 | KF114884 | KF725923 | KF725980 | KP109516 |  |
| JQ777137 | KC154025 | KC862627 | KF114885 | KF725924 | KF725983 | KP109517 |  |
| JQ777145 | KC154026 | KC862648 | KF114886 | KF725925 | KF725984 | KP109520 |  |
| JQ779074 | KC154027 | KC862666 | KF114887 | KF725926 | KF725987 | KP109521 |  |
| JQ779170 | KC154028 | KC862690 | KF114888 | KF725927 | KF725989 | KP109522 |  |
| JQ779226 | KC186127 | KC862709 | KF114889 | KF725928 | KF725990 | KP109523 |  |
| JQ779286 | KC186165 | KC862732 | KF114890 | KF725929 | KF725991 | KP109524 |  |
| JX140667 | KC186203 | KC862752 | KF114891 | KF725930 | KF725992 | KP109525 |  |
| JX140668 | KC186237 | KC862774 | KF114892 | KF725931 | KF725993 | KP109526 |  |
| JX213352 | KC186271 | KC862793 | KF114893 | KF725933 | KF725994 | KP109527 |  |
| JX213359 | KC186308 | KC862809 | KF114894 | KF725934 | KF725995 | L07426 |  |
| JX213375 | KC186349 | KC862828 | KF114895 | KF725935 | KF725996 | L22956 |  |
| JX213387 | KC186388 | KC862848 | KF145141 | KF725936 | KF725997 | L48067 |  |
| JX213405 | KC186421 | KC862858 | KF527081 | KF725937 | KF725998 | U06717 |  |
| JX213416 | KC186457 | KC862875 | KF716466 | KF725938 | KF725999 | U06718 |  |
| JX213435 | KC186498 | KC862891 | KF716467 | KF725940 | KF726000 | U06719 |  |
| JX213443 | KC186577 | KC862912 | KF725883 | KF725941 | KF726001 | U07237 |  |
| JX213454 | KC186611 | KC862931 | KF725884 | KF725942 | KF726006 |  |  |
| JX213464 | KC186650 | KC862949 | KF725885 | KF725944 | KF726007 | **KX644184 - KX644757** |  |
| JX239287 | KC186693 | KC862967 | KF725886 | KF725945 | KF726010 |  |  |
| JX239327 | KC186730 | KC862986 | KF725887 | KF725947 | KF726011 |  |  |
| JX239342 | KC186771 | KC863009 | KF725888 | KF725948 | KF726013 |  |  |
| JX845585 | KC186814 | KC863032 | KF725889 | KF725949 | KF726014 |  |  |
| JX845589 | KC186893 | KC863053 | KF725890 | KF725950 | KF726015 |  |  |
| JX845592 | KC186934 | KC863073 | KF725891 | KF725951 | KF726019 |  |  |
| JX845596 | KC186976 | KC863092 | KF725892 | KF725952 | KF726020 |  |  |
| JX845599 | KC187022 | KC863108 | KF725894 | KF725953 | KF726022 |  |  |
| JX845601 | KC187067 | KC863126 | KF725895 | KF725954 | KF726024 |  |  |
| JX845604 | KC187107 | KC863145 | KF725896 | KF725955 | KF726025 |  |  |
| JX845608 | KC187145 | KC863162 | KF725897 | KF725956 | KF770248 |  |  |
| JX845611 | KC187179 | KC863178 | KF725898 | KF725957 | KF770256 |  |  |
| JX845612 | KC187247 | KC863201 | KF725899 | KF725958 | KF770258 |  |  |
| JX972931 | KC187280 | KC863215 | KF725900 | KF725959 | KF770265 |  |  |
| JX972986 | KC187314 | KC863239 | KF725901 | KF725960 | KF770271 |  |  |
| JX973075 | KC187349 | KC863306 | KF725903 | KF725961 | KF770272 |  |  |
| JX973171 | KC187374 | KC863324 | KF725904 | KF725962 | KF770277 |  |  |
| KC148637 | KC187417 | KC863344 | KF725905 | KF725963 | KF770281 |  |  |
| KC149470 | KC187461 | KC863362 | KF725907 | KF725964 | KF770287 |  |  |
| KC154012 | KC187499 | KC863569 | KF725908 | KF725966 | KF770315 |  |  |
| KC154013 | KC187538 | KC863583 | KF725909 | KF725968 | KF770331 |  |  |
| KC154014 | KC187577 | KC863599 | KF725910 | KF725969 | KF770340 |  |  |
| KC154015 | KC187611 | KC863613 | KF725911 | KF725970 | KF770361 |  |  |
| KC154016 | KC187627 | KC863633 | KF725912 | KF725971 | KF770378 |  |  |
| KC154017 | KC187662 | KC894117 | KF725914 | KF725972 | KF770392 |  |  |
| KC154018 | KC187699 | KC894120 | KF725916 | KF725973 | KF770396 |  |  |
