# Supplementary material for: Analysis of Viral Diversity in Relation to the Recency of HIV-1C Infection in Botswana
Source: PLoS One. 2016 Aug 23;11(8):e0160649. doi: 10.1371/journal.pone.0160649 (PMC4994946; doi:10.1371/journal.pone.0160649)
Supplement: S3 Table — Table shows the results for the area under the curve (AUC) of a receiver operating characteristics (ROC) graph for the <130, <180- and <360-day cut-offs. Using only shared time-points (n = 238) significantly reduces the sample size and therefore the performance of the three assays. The performance of the three assays are therefore indistinguishable given the overlap in the confidence intervals of the AUC estimates. (DOCX) [file pone.0160649.s008.docx]

Table S3: Area under the curve (AUC) for the PwD, BED, and LAg assays for shared time-points (n=238).

| Assay | AUC | SE | 95% CI |
| --- | --- | --- | --- |
| 130-day window |  |  |  |
| PwD | 0.82 | 0.03 | 0.77 - 0.88 |
| BED | 0.82 | 0.03 | 0.76 - 0.88 |
| LAg | 0.87 | 0.03 | 0.82 - 0.92 |
| 180-day window |  |  |  |
| PwD | 0.81 | 0.03 | 0.75 - 0.87 |
| BED | 0.79 | 0.03 | 0.72 - 0.85 |
| LAg | 0.85 | 0.03 | 0.80 - 0.91 |
| 360-day window |  |  |  |
| PwD | 0.76 | 0.06 | 0.64 - 0.87 |
| BED | 0.74 | 0.06 | 0.63 - 0.86 |
| LAg | 0.76 | 0.06 | 0.65 - 0.87 |

Table shows the results for the area under the curve (AUC) of a receiver operating characteristics (ROC) graph for the <130, <180- and <360-day cut-offs. Using only shared time-points (n=238) significantly reduces the sample size and therefore the performance of the three assays. The performance of the three assays are therefore indistinguishable given the overlap in the confidence intervals of the AUC estimates.
