# Supplementary material for: Analysis of Viral Diversity in Relation to the Recency of HIV-1C Infection in Botswana
Source: PLoS One. 2016 Aug 23;11(8):e0160649. doi: 10.1371/journal.pone.0160649 (PMC4994946; doi:10.1371/journal.pone.0160649)
Supplement: S4 Table — Table shows the estimated Mean Duration of Recent Infection (MDRI), average time ‘recent’ while infected for less than some time cut-off T for the BED, LAg and PwD assays. (DOCX) [file pone.0160649.s009.docx]

Table S4: Mean Duration of Recent Infection for BED, LAg and PwD Assays.

| Assay (cut-off) | No. of specimens | *T* (days) | MDRI (95% CI) |
| --- | --- | --- | --- |
| BED (0.8 ODn) | 554 | 730.5 | 267 (212 – 335) |
| LAg (1.5 ODn) | 579 | 730.5 | 129 (81 – 190) |
| PwD (0.005) | 238 | 730.5 | 128 (92 – 185) |

Table shows the estimated Mean Duration of Recent Infection (MDRI), average time ‘recent’ while infected for less than some time cut-off T for the BED, LAg and PwD assays.
